# High resolution seismic data coupled to Multibeam bathymetry of Stromboli island collected in the frame of the Stromboli geophysical experiment: implications with the marine geophysics and volcanology of the Aeolian Arc volcanic complex (Sicily, Southern Tyrrhenian sea, Italy)

**DOI:** 10.1186/2193-1801-3-232

**Published:** 2014-05-08

**Authors:** Gemma Aiello, Vincenzo Di Fiore, Ennio Marsella, Salvatore Passaro

**Affiliations:** Institute of Marine and Coastal Environmental Area (IAMC), National Research Council of Italy (CNR), Calata Porta di Massa, Porto di Napoli, 80133 Naples, Italy

**Keywords:** Stromboli volcano, Aeolian arc, Multibeam bathymetry, Seismic stratigraphy, Volcanology, Southern Tyrrhenian sea

## Abstract

New high resolution seismic data (Subbottom Chirp) coupled to high resolution Multibeam bathymetry collected in the frame of the Stromboli geophysical experiment aimed at recording active seismic data and tomography of the Stromboli Island are here presented. The Stromboli geophysical experiment has been already carried out based on onshore and offshore data acquisition in order to investigate the deep structure and the location of the magma chambers of the Stromboli volcano. A new detailed swath bathymetry of Stromboli Island is here shown and discussed to reconstruct an up-to-date morpho-bathymetry and marine geology of the area compared to the volcanologic setting of the Aeolian Arc volcanic complex. Due to its high resolution the new Digital Terrain Model of the Stromboli Island gives interesting information about the submerged structure of the volcano, particularly about the volcano-tectonic and gravitational processes involving the submarine flanks of the edifice. Several seismic units have been identified based on the geologic interpretation of Subbottom Chirp profiles recorded around the volcanic edifice and interpreted as volcanic acoustic basement pertaining to the volcano and overlying slide chaotic bodies emplaced during its complex volcano-tectonic evolution. They are related to the eruptive activity of Stromboli, mainly poliphasic and to regional geological processes involving the intriguing geology of the Aeolian Arc, a volcanic area still in activity and needing improved research interest.

## Introduction

New high resolution seismic data (Subbottom Chirp) coupled to high resolution Multibeam bathymetry collected in the frame of the Stromboli geophysical experiment aimed at recording active seismic data and tomography of the Stromboli island are here presented and interpreted to improve the geologic and volcanologic knowledge of the Tyrrhenian offshore around the Stromboli volcano.

**Figure 1:**
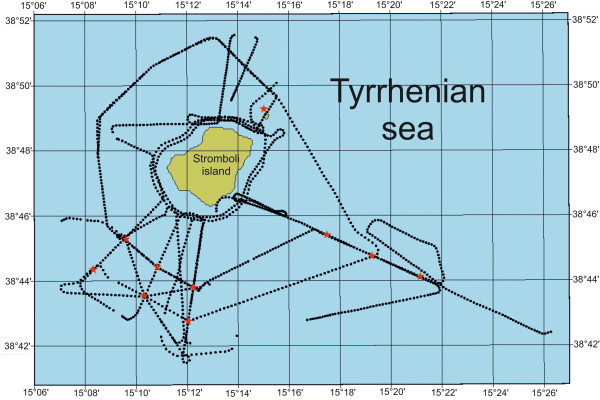
**Navigation map of seismic profiles recorded in the oceanographic cruise STRO-06 of the Stromboli island.**

Preliminary results on the Stromboli geophysical experiment, carried out based on onshore and offshore data acquisition around the Stromboli volcano and finalized to reconstruct a seismic tomography of the volcano and to investigate the deep structure and the location of the magma chambers have been already shown (Marsella et al. 2007a, 2007b; Castellano et al. 2008). A detailed swath bathymetry around the Stromboli volcano has been recorded and is here presented during the oceanographic cruise STRO-06 (R/V Urania; December 2006). Subbottom Chirp profiles have also been acquired according to a largely spaced grid perpendicular and radial with respect to the volcanic edifice (Figure 1).

During the Stromboli geophysical experiment, wide-angle refraction seismics was performed all around the Stromboli volcano by a 4 GI-GUN array (property of CNR-IAMC, Naples, Italy). The permanent seismic network of the National Institute of Geophysics and Oceanography (INGV, Italy) has been used, incremented for the experiment of 18 temporary stations and 18 Ocean Bottom Seismometers (OBS), deposited on the southeastern, southwestern and northeastern submerged flanks of the volcano after detailed morphobathymetric analysis (Di Fiore et al. 2006; Marsella et al. 2007a; Castellano et al. 2008). Due to its high resolution, the new DTM of the Stromboli island, here presented gives interesting information about the submerged structure of the volcano, particularly about the volcano-tectonic and gravitational processes involving the submarine flanks of the edifice.

## Geodynamic setting of the southern Tyrrhenian basin

### Evolution of the Tyrrhenian-Apennines system

**Figure 2:**
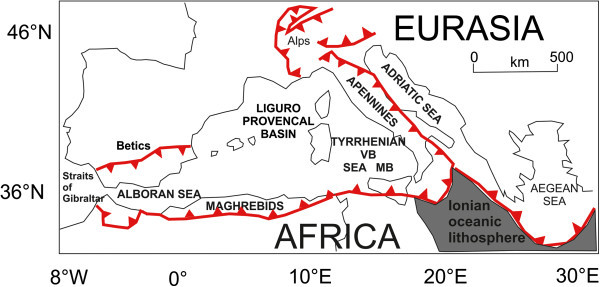
**Structural sketch map of the Mediterranean area (modified after Marani and Trua** 2002 **).** Red lines with filled triangles represent thrust fronts. Key. VB: Vavilov Basin. MB: Marsili Basin.

The irregular conformation of the African and Euro-Asiatic continental margins, with the interposed Adriatic microplate (Panza et al. 1980; Pontevivo and Panza 2002) has given very complex the geodynamics of the Mediterranean region (Figure 2). In the Middle-Late Cretaceous (about 80 My B.P.) the opening of the Atlantic Ocean has determined an inversion of the relative movement of the African plate with respect to the Eurasiatic one and has produced as a main result the closure of the Thetys ocean. The process of closure ended in the Eocene with the Alpine orogenesis (Dewey et al. 1973).

Starting from the Oligocene the phases which have determined the present-day configuration of the western Mediterranean reflect a progressive migration of the Adriatic-Ionian lithosphere in the frame of a complex subduction system, migrating westwards (Malinverno and Ryan 1986; Figure 2). From the end of the Oligocene and the beginning of the Miocene (about 26 My B.P.) the extensional phases, to the west of the trench in the back-arc area, have produced a rifting of the Ercinian crust of the European foreland, which has given origin to the opening of the Ligurian-Provenzal Ocean, with a counterclockwise rotation of about 25°-30° of the Sardinia-Corsica block, happened from 30 and 16 My B.P. (Van der Voo 1993). During this period the first compressional phases of the Apenninic orogenesis verified, during which the more internal tectonic units moved eastwards overlying the outermost ones. In the Early-Middle Tortonian (about 12 My B.P.) another compressional tectonic phase verified, during which the uplift of part of the central Apennines happened.

The opening of the Tyrrhenian basin started from the Late Tortonian with a process of rifting which began along a N-S direction next to the eastern margin of the Sardinia-Corsica block (Lavecchia 1988; Sartori 1989). During the Plio-Pleistocene, the extensional process migrated eastwards superimposing, during space and time, to the compressional tectonics involving the Apenninic nappes during their progressive advance towards the Adriatic foreland. During recent times the Apennines thrust and fold belt is in compression along the outer front, while along the axis of the chain prevail extensional faults, whose dislocations have generated the most important earthquakes of the Apenninic ridge (Istituto Nazionale di Geofisica e Vulcanologia 2005).

**Figure 3:**
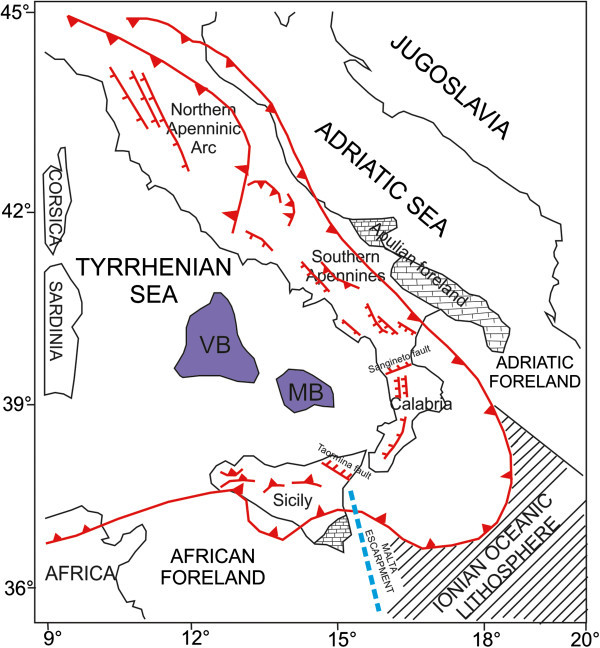
**Sketch map of the main structural elements of the Italian Peninsula (modified after Marani and Trua** 2002 **).** The line with the white triangles represents the outer front of the Apenninic-Maghrebide orogenic system. The line with the black triangles traces the continuous overthrusting along the northern Apenninic Arc. The undulated lines indicate the main extensional faults which characterize, starting from the Pleistocene the southern Apennines and the Calabrian Arc. The dotted areas represent the Apulian foreland (located in the Puglia region) and the African foreland (located in the northern Siciliy). On the map the main hypothesized tectonic lineaments have been also represented: TAO – Taormina line; SAN – Sangineto Line;VB: Vavilov Basin; MB: Marsili Basin (modified after Marani and Trua 2002).

From the Pliocene to the Pleistocene a set of compressional phases has accompanied the superimposition of the most inner tectonic units on the outer sectors of the Adriatic foreland. The orogenesis has determined a large scale flexure, gradually migrating eastwards, with the consequent formation of the Apenninic foredeep, consequently involved in the processes of thrusting. The present-day foredeep is named the Padan-Bradanic foredeep and is located on continental crust and includes Plio-Quaternary sediments for an overall thickness of 9 km (Royden 1993). The Apenninic orogenesis has produced a crustal shortening along a E-W direction, not uniform along all the chain. This different kinematics has determined a not uniform curvature of the Apenninic ridge, which can be distinguished in two sectors (Figure 3): the northern Apennines showing a concavity towards the Tyrrhenian sea, extends from the Monferrato to the Latium-Abruzzi region; the southern Apennines, from the Molise-Abruzzi region to the Basilicata region, which doesn’t show an evident curvature and the Calabria-Peloritani Arc, with a greater curvature and concavity towards the Tyrrhenian sea (Patacca et al. 1989; 1990; Doglioni 1991).

The Northern Apennines represent an anomalous zone with respect to the Southern Apennines, since it had a different evolutionary zone. In fact, its genesis is related to the Alpine orogenesis, for the most part included among the Sangineto fault and the Soverato-Capo Vaticano fault and to the evolution of the Maghrebids, for the sector between the Soverato-Capo Vaticano fault and the Sicily (Boccaletti et al. 1990). The northern and southern sectors are put in contact along a tectonic lineament, the Ortona-Roccamonfina line, which represents a boundary between two domains with a different kinematics.

### The opening of the Tyrrhenian sea

Regarding the evolution of this part of the Mediterranean several geophysical and geodynamic models have been purposed, which evidence the complex evolution of this sector.

The extensional phases, started about 30 My B.P., in the Ligurian-Provenzal basin and then continued in the Tyrrhenian basin, happened in an episodic manner in a general setting of continental collision. The two extensional phases are separated among them by about 5 My B.P. and their opening is strictly linked to the African lithosphere subducting towards W-NW.

In the Central Mediterranean the first evidence of the subduction of the Adriatic microplate started at the beginning of the Paleocene (about 60 My B.P.) with the occurrence of flysch deposits and high pressure and low temperature metamorphism (Jolivet et al. 1998). During the Oligocene (about 32 My B.P.) the first indications on the geodynamic evolution of the area come when volcanic arcs form in the area Sardinia-Provence: the extension starts at the back of the Apenninic accretion wedge (Beccaluva et al. 1989).

Between the Oligocene and the Early Miocene it begins a rifting phase, starting to the formation of the Ligurian-Provenzal Basin and to the rotation of 25°-30° in a counterclockwise sense of the Sardinia-Corsica block with an average velocity of extension of the back-arc basin of about 3–4 cm/year (Van der Voo 1993).

**Figure 4:**
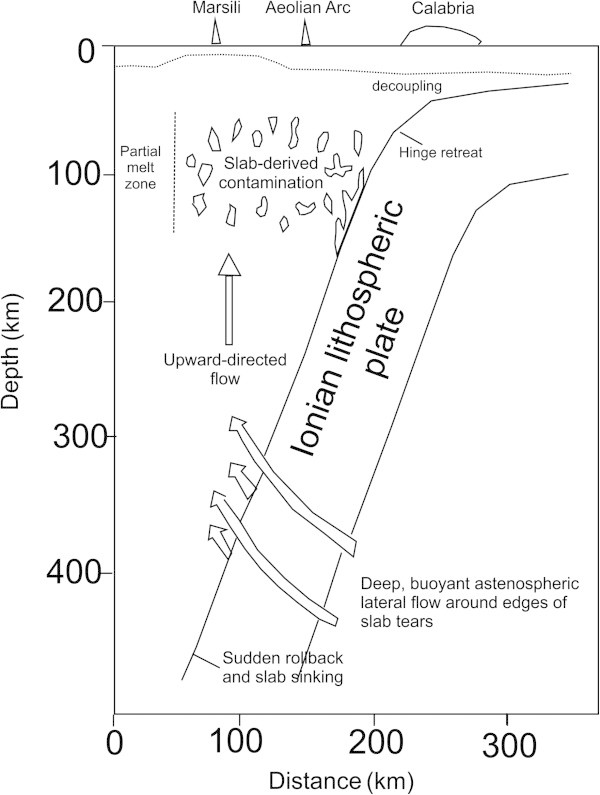
**Sketch map showing the subduction of the Ionian lithosphere (modified after Marani and Trua** 2002 **).** The location of the Marsili ridge, Aeolian Arc and Calabria has been also indicated. An abrupt increase of the rollback due to the full development of lateral tears in the Ionian slab, between Early and Middle Pleistocene, generates the lateral flow of deep asthenosphere around the slab margins. The dotted line corresponds to the crustal thickness of the Tyrrhenian plate.

The extension and the subsidence happened during the Late Tortonian sign the beginning of a new episode of expansion. The lithospheric rifting causes the separation of the Calabria block and begins the opening of the southern Tyrrhenian basin leaving at its back the Sardinia and Corsica blocks. After about 5 My B.P., contemporaneously to the lateral shift of the Calabria block, a new process of rifting starts, testified by the syn-rift deposits in Sardinia and Calabria (Sartori 1989; Sartori et al. 1989). New oceanic crust forms into two separate basins (Bigi et al. 1989), first in the Magnaghi-Vavilov basin (4–5 My B.P.) and then in the Marsili basin (< 2 My B.P.). These ones are considered as small back-arc basins, being linked to the occurrence of the Ionian lithospheric slab, subducting towards NW (Marani and Trua 2002; Figure 4).

After the development of these back-arc basins, the volcanism of the southern Tyrrhenian basin is migrated from W to SE, from the Sardinia to the present-day Aeolian Arc (Serri 1997; Kastens et al. 1988; Bigi et al. 1989; Sartori 1989; Savelli, 2001), developing the present-day configuration of the Marsili back-arc basin. The average rate of opening of the southern Tyrrhenian basin during the last 5 My B.P. is of about 6 cm/y (Malinverno and Ryan 1986; Patacca et al. 1990).

During present times the lithospheric structure shows two thinned regions of oceanic crust (30–40 km) separated by lithosphere relative to the Sardinia continental block thick about 80 km (Faccenna et al. 2001); moreover, the basin is characterized by a crustal thinning, which reduce more and more up to less than 10 km in the sector towards SE (Panza et al. 2003).

The correlation of the data on the lithospheric thickness with those ones regarding the distribution of the heat flow in the Tyrrhenian region (Erickson 1970; Hutchinson et al. 1985; Mongelli 1991; Mongelli and Zito 1994) has evidenced that the Tyrrhenian sea can be divided into three parts: the northern Tyrrhenian sea, the central Tyrrhenian sea and the southern Tyrrhenian sea. The southern Tyrrhenian sea is the widest and thinnest part and shows the deeper parts of the basin (more than 3500 m). To the south-east of this area the highest values of heat flow have been recorded (Morelli 1970). The central part of the Tyrrhenian sea shows the lowest values of the heat flow (Zito et al. 2003). In the basin located northwards, in proximity to the Tuscany, the heat flow values are very high (160 mw/mq).

This trending of the heat flow seems to be linked to the active magmatism occurring in the Tyrrhenian basin and then to the subduction rate of the lithospheric slab: in fact, in the part of the Calabrian Arc where there is a faster retreating, a strong extension of the lithosphere may be observed, coupled with high heat flows. On the contrary, in the southern Apennines, where the retreating of the subduction system is very low, active volcanism is lacking, extension rate is minimum and the heat flow is low. In the central-northern Apennines, where the subduction is faster, there are active seismicity and magmatism in Latium and Tuscany and high heat flows.

### Geodynamic models on the extension of the Tyrrhenian sea

In order to explain the geodynamic evolution of the Mediterranean area and in particular, of the Tyrrhenian-Apennines system several evolution models have been purposed, through which the observed structural complexities should be explained.

The main aspects which have to be considered in a general model are the relative movements between the African and European plates, the coexistence of compressional and extensional regimes, respectively on the outer and inner fronts of the Apenninic chain, the eastward migration of the above mentioned tectonic regimes, the distribution of the seismicity along the Apenninic Arc, the geochemical and petrological characteristics of the Tyrrhenian and peri-tyrrhenian magmatism, the thermal flux and the palaeomagnetic evidence.

**Figure 5:**
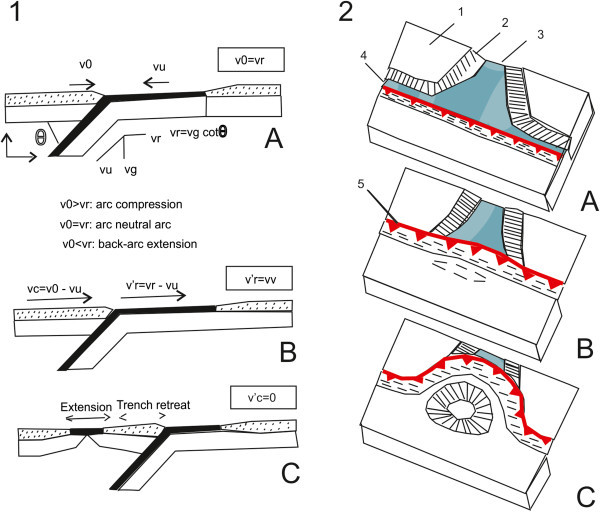
**Sketch diagram according to the geodynamic model of Malinverno and Ryan** 1986 **(modified after Malinverno and Ryan** 1986 **).** 1: the evolution of the arc-trench system **(A)** is related to the velocity of the slab retreatment (Vr) and to the dipping angle (θ). In a fixed reference system with respect to the subducting plate **(B)** the velocities which determinate the tectonic style are: vc (velocity of convergence among plates), v’r (velocity of retreatment of the overthrusting plate). When the convergence does not exist (vc = 0) it verifies extension in the overthrusting plate in order to balance the retreatment of the subduction **(C)**; 2) development of a basin, reflected by the outwards arc migration. 1: continent; 2: continental margin; 3: oceanic sector; 4: active subduction zone; 5: subduction zone not still active. The stages **A**, **B** and **C** indicate the time evolution.

The geodynamic model developed by Malinverno and Ryan (1986) implies that the Tyrrhenian-Apennines system evolved in relationships to the westward subduction of the Adriatic-Ionian lithosphere. The arc-trench system should progressively migrate eastwards (roll-back) due to the sinking of the subducting plate. In this model two main assumptions exist, the first one that the lithospheric plates have a plastic behavior, the second one that the evolution of a subduction zone is linked to the ratio between the velocity of convergence among the plates and the velocity of retreatment of the overthrusting plate. The geodynamic evolution of the Tyrrhenian-Apennines system should then triggered by the southeastwards retreating of the subduction zone **(**Figure 5; Malinverno and Ryan 1986). The described model explains some most characteristic aspects of the Tyrrhenian-Apennines area, such as the radial geometry of the Apenninic and Maghrebide chains with respect to the Tyrrhenian basin, the contemporaneous verification of a compressional regime stress on the outer front of the chain and extensional in the back-arc zone (Frepoli and Amato 1997), the subduction under the Calabrian Arc (Amato et al. 1997) and finally, the calcalkaline composition of the Aeolian volcanism (Barberi et al. 1974; Beccaluva et al. 1994). In fact, the geochemical data demonstrate that the magmatism of the Aeolian islands is compatible with a mantle having a composition between MORB (mid-oceanic ridge basalt) and OIB (oceanic island basalt) enriched of fluids and in some cases also of sediments derived from a subducted oceanic crust.

Another geodynamic model of the Tyrrhenian-Apennines system has been purposed by Doglioni et al. (1999), based on an active subduction in the orogenic system. According to this model, the Tyrrhenian sea is interpreted as a back-arc basin, but its asymmetric shape could be the expression of an astenospheric push on a laterally heterogeneous lithosphere. The lithospheric flux proceeding eastwards is responsible of both the retreating in the same direction of the arc-trench system and of the uplift along the Apenninic chain.

### Seismicity of the Tyrrhenian-Apennines system

The seismicity of the Italian Peninsula is characterized mainly by a shallow seismicity under the Apennines, a region of deep crustal seismic events eastwards of the Apenninic chain and sub-crustal earthquakes under the northern Apennines and the Calabrian Arc (Chiarabba et al. 2005). As a consequence, three main sectors, well distinguished from a seismo-tectonic point of view, may be defined, i.e. the Northern Apennines, the Southern-Central Apennines and the Calabrian Arc (Amato et al. 1997).

The Northern Apennines has a seismicity with maximum hypocentral depths reaching the 90–100 km (Amato et al. 1997). The verification of sub-crustal earthquakes, with epicenters located northwards of the 43° of latitude and to the south-west of the chain axis, has suggested the occurrence of a subducting plate under the northern Apennines (Selvaggi and Amato 1992). The crustal seismicity is widespread along all the orogenic axis and occurs in the inner part of a belt wide about 50–70 km perpendicular to the chain, corresponding to the maximum topographic heights (Amato et al. 1997).

In the Southern-Central Apennines there is only a crustal seismicity, related to extensional structures (Amato and Montone 1997) and concentrated along a belt, wide about 30–50 km and NW-SE trending (Amato et al. 1997).

In the Calabrian Arc a sub-crustal seismicity has been mainly recorded with hypocenters reaching 450 km of depth (Giardini and Velonà 1991; Selvaggi and Chiarabba 1995; Amato et al. 1997). On the other side, the shallow seismicity shows events with a magnitude greater than 4.5 and a component of compressional deformation localized along the northern coast of the Sicily. The shallow events are clearly confined to the west of the Aeolian Archipelago and appear scattered and rare in the western part, while the intermediate and deep seismicity is confined to the west of the Aeolian islands (Pondrelli and Piromallo 2003). The seismicity of the last twenty years shows the existence of a subduction of oceanic lithosphere under the Calabrian Arc; it defines a Benioff plan thick 40–50 km, wide 200 km and NW dipping with an angle of 70° up to depths of 400 km (Holcomb 1989; Amato et al. 1997; Lucente et al. 1999; Chiarabba et al. 2005).

### Tomographic interpretation of the southern Tyrrhenian basin

The Southern Tyrrhenian basin has been the subject of many tomographic studies aimed at reconstructing the lithospheric structure under the sea. These types of seismological images allow to define the velocity anomalies of the P waves under the study area, translating in the spatial definition of the lateral heterogeneities existing in the lithosphere and in the upper mantle in terms of fast and slow zones with respect to a reference velocity model. The first ones are interpreted as colder and denser materials with respect to the surrounding environment and usually associated with subducted lithospheric remnants; the second ones are instead interpreted as hotter and less dense bodies, which are typical expressions of melted volcanic materials or, at sub-crustal depths, of astenospheric fluxes (Hirahara and Hasemi 1993; Piromallo and Morelli, 1997).

All the tomographic models have evidenced a high velocity zone, interpreted as the subducting Ionian slab NW dipping (Cimini 1999, 2004; Lucente et al. 1999; Montuori 2004). The slab shows an evident vertical continuity with a high immersion angle (70°-75°) in the first 400 km of depth, where it is concentrated the most part of the deep seismicity and becomes sub-horizontal at greater depths. In the model of Montuori (2004), obtained by using for the first time teleseismic events recorded by submarine stations, is well reconstructed the lateral extension, which results to be of 200 km under 150–200 km of depth, while it reduces to 100 km at shallower depths.

**Figure 6:**
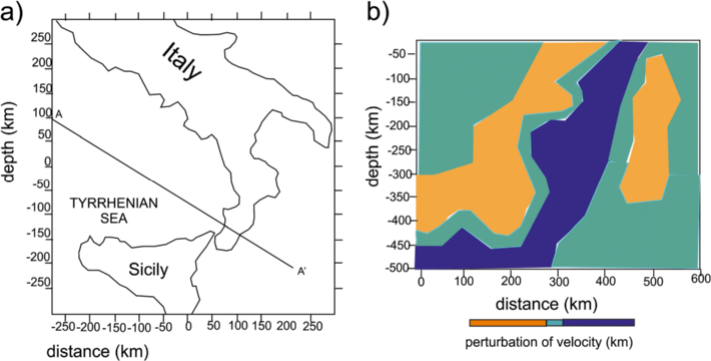
**Tomographic profile across the Aeolian island (modified after Montuori** 2004 **) a: AA’ profile across the Aeolian island; b: perturbations of velocity anomalies across the AA’ profile.** It is worth noting the occurrence of a high velocity continuous body, reaching values of perturbations of 5%, NW dipping with an angle of 70°-75°, up to 400 km; under this depth the body assumes a sub-horizontal shape. The seismic events (yellow dots) are reported on the section if they happen since 50 km from the projection plan. The deep seismicity is concentrated in the inner of this structure. Zones of pronounced low velocity are well evident along all the model and border the whole fast structure.

The high velocity zone is laterally surrounded by low velocity zones in all its vertical extension, which are interpreted as astenospheric fluxes next to the subducting plate, or as the trace of convective cells determined by the subduction (Figure 6). Moreover, the correspondence between the low velocity zone and the Aeolian Arc indicates that the subduction has generated the volcanism observed in the region (Montuori 2004).

### The Aeolian islands

The Aeolian Arc is a volcanic structure, long about 200 km located in the inner margin of the Calabrian-Peloritan Arc. The arc is formed by seven emerged volcanic edifices (Alicudi, Filicudi, Salina, Lipari, Vulcano, Panarea and Stromboli) and by several submarine volcanoes surrounding the Marsili basin. The structural elements and the volcanic activity in the area allow to identify three distinct sectors: the western sector (Alicudi and Filicudi); the central sector (Salina, Lipari and Vulcano) and the eastern sector (Panarea and Stromboli; Marani and Gamberi, 2004a, 2004b; Bortoluzzi et al. 2010).

The oldest volcanic activity of the Aeolian Arc is dated back at 1.3 My B.P. and conceals the Sisifo submarine volcano, located in the western sector of the archipelago. Now the only emerged volcanoes which may be considered still active are Stromboli, Vulcano and Lipari.

The type of volcanism of the Aeolian Arc is of convergence between plates and the islands pertain to an arc-trench system, resulted from the collision between the African and Eurasiatic plates, with the occurrence of a subducting slab NW dipping under the Tyrrhenian sea (Barberi et al. 1974).

In the Aeolian volcanism, active from about one million of years, two eruptive phases separated by a period of quiescence in the Late Pleistocene may be distinguished (Keller 1974). During the first phase the islands of Alicudi, Filicudi, Panarea, Lipari and Salina have been formed. During the second phase the completion of these latter ones happened and the birth of Vulcano and Stromboli (Barberi et al. 1974).

Regarding the chemical composition of the erupted products, a time evolution may be observed which can be resumed in three series with increasing contents of potassium (Barberi et al. 1974). They are the calcalkaline series (basalts rich in Al and dacites), corresponding to the oldest products, the andesitic series, rich in K (Lipari and Stromboli) and the shoshonitic series (shoshonitic basalts and rhyolites), regarding the most recent volcanic products (Vulcano, Lipari and Stromboli).

A magmatic chamber at 2 km of depth is located under the sea bottoms separating Lipari from Vulcano and the constant low values of the Sr isotopic rate indicate that the magmas are sub-crustal in provenance and not involved by phenomena of crustal contamination. The shoshonitic nature of the most recent volcanoes indicates, moreover, that the arc is in its latest stages of evolution (Keller 1974; Sigurdsson 2000; Vidal and Merle 2000).

The nature of the submarine volcanoes is compatible with the hypothesis of an expanding marginal basin; in fact, they are composed of basaltic volcanoes, probably related to extensional fractures having a NW-SW trending, with tholeitic products at their base and alkaline products in the highest parts (Barberi et al. 1974).

#### Stromboli

**Figure 7:**
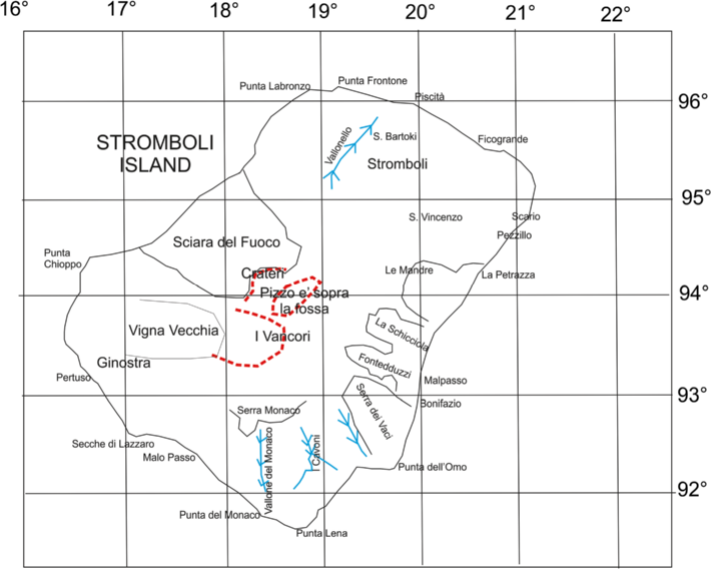
**Location map of the Stromboli island, reporting main localities of the volcanic island onshore.** The red lines indicate the main crateric rims of the Stromboli volcanic edifice; the black lines indicate the main reliefs of the island; the blue lines indicate onshore drainage axes. The location of the Sciara del Fuoco lava flow has also been indicated.

Stromboli is the northest island of the Aeolian archipelago and has an area of 12.6 km^2^. The volcanic edifice, localized at a latitude of 38.8°N and a longitude of 15.0°E, emerges of 924 meters above the sea level and extends up to 3000 m under the sea level. Its top is composed of two crests having a half-moon shape: the outer one is named “I Vancori”, while the inner one is known as “Pizzo sopra La Fossa” (Figure 7). Both the crests are the remnants of old volcanic edifices. The erosion has carved deep canyons along the slopes of these old edifices and a wide slope covered by the ashes of the recent activity extends from the upper part of the volcano to the south-eastern one. The main onshore drainage system have also been indicated in the sketch map of Figure 7. The active craters are not located at its top, but at 100–150 m under Pizzo Sopra La Fossa in a depression formed 5000 years B.P. as a consequence of the collapse of a part of the volcanic edifice (Figure 7). The craters, still characterized by volcanic activity are now three and their set is defined as a terrace, a structure continuously changing and in gradual increasing upwards. The lava flows run towards a large valley dipping seawards, up to water depths of 1700 meters (Romagnoli et al. 1993). It is located on the NW side of the volcano and is named Sciara del Fuoco and was formed due to large rock falls and flows (Pasquarè et al. 1993; Figure 7).

The emerged part of the Stromboli strato-volcano is mainly formed during two distinct cycles of activity (Barberi et al. 1974). The old cycle is composed of rhythmic eruptions of pyroclastic materials and lava flows, giving rise to the formation of all the eastern sector of the island. The recent cycle, during which lava flows have been mainly erupted, is responsible for the formation of all the western half of the Stromboli island. Now the recent cycle continues with the activity of the craters surrounding the Sciara del Fuoco lava flow.

Stromboli is one of the few volcanoes on the earth which has a continuous eruptive activity with periods of few days or decades lasting for about 2000 years. The persisting activity implies a magmatic chamber of great dimensions supplied with continuity and mainly consisting in explosions of moderate energy (Barberi et al. 1993). The explosions last up to 10 seconds and repeat at intervals of about 10–20 minutes and are connected to fluvio-dynamical movements of the magma in the inner of the conduit (Ereditato and Luongo 1997; Chouet et al. 1999; Ripepe et al. 2001; De Martino et al. 2004). They involve a set of fragments in the order of 10^2^-10^4^ kg (Chouet et al. 1974; Ripepe et al. 1993) and a gas volume in the order of 10^3^ m^3^ (Chouet et al. 1974). The sources of these explosions are concentrated at a depth of 200 m about under the top of the craters (Chouet et al. 1997; Saccorotti and Del Pezzo 2000). This moderate activity has been interrupted by episodes of greater entity, accompanied by lava fluxes happened in the 1975 (Capaldi 1978), in the December of 1985 (De Fino et al. 1988), in the 1993 (Bonaccorso et al. 1996) and the last in the December 2002 (Bonaccorso et al. 2003).

The last eruption of the Stromboli, started on the 28 December 2002, has been anticipated by an increase of the explosive activity starting from the May 2002 and by the increase of lavas in the craters from November, when from the northern flank of the second crater a small lava flow erupted, propagating in the upper part of the Sciara del Fuoco. Starting from December the explosions and the heights of the eruptions was particularly intense in the first crater, located to the NW, up to reach the 200 m with respect to the crater. The same day, the activity ended with the opening of an eruptive fracture NE trending, long about 300 m. The opening of the fracture caused a shifting towards NW of the upper part, located to the SE of the Sciara del Fuoco and the formation of small slopes parallel to the direction of the slope originated by the landslide which reached the sea generating a tsunami (Bonaccorso et al. 2003).

## Previous marine geological studies

Many marine geological studies have been produced aimed at reconstructing the slope failures, deep sea deposits and other volcanological and geological aspects concealing the Stromboli volcano. Some of them are enclosed in a book dealing with the 2002–2003 Stromboli eruptions (Baldi et al. 2008; Bertagnini et al. 2008; Bonforte et al. 2008; Calvari et al. 2008; Marani et al. 2008; Martini et al. 2008; Ripepe et al. 2008; Tibaldi 2001; Tibaldi 2008; Tinti et al. 2008; Tommasi et al. 2008).

On the December of the year 2002, following 17 years of an intense strombolian activity and gradual increasing in the frequency of explosions within the upper craters of the volcano, a long eruptive fissure opened in the north-eastern flank of the Stromboli volcano (Calvari et al. 2008). The explosive activity fed hot avalanches flowing down the Sciara del Fuoco towards the Tyrrhenian sea, followed by an intense emission of lavas. After a few days the fractures formed along the Sciara del Fuoco caused the failure of two large portions of the NE unstable slope of the volcano (Tommasi et al. 2008). The landslides triggered two tsunami waves extending over 100 m inland, reaching the town of Milazzo and the northern coast of Sicily (Tinti et al. 2008). The sequence of landslides occurring soon after the eruption and invoving the NW flank of the volcano has been reconstructed (Tommasi et al. 2008). The landslides involved the north-eastern part of the Sciara del Fuoco slope, producing tsunami waves along the coasts of the island. The volumes of the masses detached from the subaerial and submarine slopes have been quantified to compare the pre-slide and the post-slide surfaces, obtained through aerophotogrammetric and bathymetric data in order to reconstruct the geometry and the kinematics of landslides (Tommasi et al. 2008). The integrated subaerial and submarine morphological evolution of the Sciara del Fuoco after the 2002 landslide has been reconstructed through digital photogrammetry and Multibeam bathymetry to obtain high resolution digital elevation model of land and sea-floor surface of the NW flank of Stromboli (Sciara del Fuoco depression; Baldi et al. 2008). The merging of subaerial and submarine data and the comparison of several Digital Elevation Models has allowed the estimate of the mass volumes involved in the failures and the monitoring of the morphological changes induced by erosional and depositional processes of the volcano (Baldi et al. 2008). The map of residuals obtained by subtracting the pre-and-post-slide digital models have shown that in its subaerial portion the slide is articulated in two different slabs. At the same time, shallow bathymetric surveys have revealed a wide sub-circular slide scar and over 45 m deep, related to the tsunami event. The comparison of pre-and-post-slide DEMs let to know that the materials mobilized by the submarine slide is of about 9.3 × 10^6^ m^3^ (Baldi et al. 2008; Chiocci et al. 2008a, 2008b; Tommasi et al. 2008).

The submarine morphology of the Sciara del Fuoco valley has been reconstructed in detail (Marani et al. 2008). Two submerged scarps delimit the shallower portion of a broad valley, having a flat bottom. Beyond the 900 m of water depth the deep water eastern margin of the Sciara del Fuoco valley consists of an incision connected to the eastern margin that arches in a northward direction down to 1700 m of water depth, diminishing in relief with depth. The landslide deposit consists of a proximal coarse-grained landslide deposit on the volcano slope and of a distal sandy turbiditic deposit (Marani et al. 2008). The proximal deposit includes two facies; the first one is a chaotic coarse-grained deposit, while the second one is a sandy facies developing laterally over the coarse-grained deposit.

Several marine surveys were carried out offshore the Sciara del Fuoco valley in order to monitoring the Stromboli submarine slopes after the December 2002 landslide (Chiocci et al. 2008a). The morphological changes and the depositional processes leading to the gradual filling of the slide scar have been studied in detail. The slide scar has been progressively filled with lava and volcaniclastic debris. During the first month and half the filling rate was very high due to the entrance of lava flows into the sea and to the morphological change of the slope; during the following months the filling rate decreased when the eruptive vents moved upwards and the volcanic eruption stopped. After four years (February 2007) the half of slide scars were filled, but a new eruption occurred and a lava delta was constructed in the 2002 scar, influencing the natural change of the slope. The morphological reconstruction of geometry and volume of scar filling during the period 2002–2007 evidenced a punctuated and fast shift of the depocenters and the emplacement of debris deposits, quickly fossilizing the landslide (Chiocci et al. 2008a).

High resolution bathymetric and backscatter maps offshore the Stromboli island have been presented, coupled with a geological interpretation of their volcanic, structural and sedimentary features (Bosman et al. 2009). The volcanic edifice is characterized by a sub-conical shape, symmetric with respect to a NE-SW axis. The dimensions of the Strombolicchio volcano, located to the NE of the Stromboli island, have been reconstructed by redrawing its morphology before the wave erosion. On the north-eastern submarine flank of Strombolicchio, a N64°E structural trend controls the shape of the Strombolicchio canyon (Bosman et al. 2009). On the southern side of Stromboli, the submarine flank has a radial structural trend, possibly reflecting a volcanic stress regime. Large-scale lateral collapses have affected both the NW and the SE flanks of the volcano, producing large debris avalanche deposits (Bosman et al. 2009).

New detailed swath bathymetry and Sidescan Sonar data collected on the submerged flanks of Stromboli, integrated with seismic data and seabed sampling indicate that repeated lateral instability processes occurred on the eastern flank of the volcano, although no debris avalanche deposits were known before the high resolution exploration of the seabed (Romagnoli et al. 2009). This flank of the island is opposite to the north-western side, affected by repeated flank collapses and this setting is evident of a structurally-controlled instability of the flanks of the volcanic edifice. Two large scale lateral collapses are evidenced by a block field, cropping out on the middle-lower eastern submerged slope and by a chaotic unit actually embedded within the volcaniclastic sequence at the foot of the submerged flank. A morphological continuity can be envisaged between this submerged scar and the inferred subaerial one. A spatial and temporal reconstruction of possible events is also proposed. The chaotic debris avalanche unit, buried within the volcaniclastic apron at the slope base and partially reworked in its distal part within the Stromboli canyon floor is thought to be the result of a lateral collapse event. The megablock field can instead result from a more superficial debris avalanche (Romagnoli et al. 2009).

Submarine portions of Stromboli volcano account for about 98% of the whole extent of the volcanic edifice and are mostly covered by volcaniclastic sediments that made up a modern volcaniclastic apron (Casalbore et al. 2010). This apron shows a large variability both across and along slope of morphologies and deposits related to mass wasting and reworking processes, passing into areas covered by hemipelagic sedimentation. A large spectrum of erosional and depositional features was recognized on the surface of the apron. On the submerged shelves, shore platforms and depositional terraces submarine features related to wave action and sea level fluctuations are present, acting for the storage and reworking for the volcaniclastic materials derived from the subaerial portions. Turbidity currents acted on the slope, generating erosional furrows and throughs, channel-levee complexes and coarse-grained sediment waves (Casalbore et al. 2010).

The most active area of the apron lies offshore the Sciara del Fuoco, on the north-western flank of the volcanic edifice, where a large amount of coarse-grained volcaniclastic material is deposited by turbidity currents. Distinct volcano-sedimentary areas of the edifice have been distinguished (Casalbore et al. 2010). The NW and SE portions of the Stromboli island are characterized by the emplacement of wide and thick debris avalanche deposits, related to large scale sector collapses, representing the most important mass wasting processes making up the apron. Such a deposits are interstratified within different volcaniclastic sequences and can be eroded or fossilized by successive gravity flows. The result is a complex succession of facies, where it is often difficult to depict an arranged series of processes. On the contrary, the SW and NE flanks of Stromboli island are characterized by a more ordered evolution of processes and deposits making up the apron, giving rise to a less complex stratigraphic architecture (Casalbore et al. 2010).

## Data and methods

The oceanographic cruise STRO-06 was carried onboard of the R/V Urania of the National Research Council of Italy (Marsella et al. 2007a; Castellano et al. 2008). This ship is normally used for geologic, geophysic and oceanographic work in the Mediterranean sea and adjoining waters. The R/V Urania is equipped with DGPS positioning system, single-beam and Multibeam bathymetry and integrated geophysic and oceanographic data acquisition systems, other than water and sediment sampling. Additional equipment can be accommodated on the keel or towed, like Sidescan Sonars.

### Navigation and positioning

The vessel was set up for Multibeam data acquisition and navigation by using the PDS2000 software by RESON. The UTC absolute time was measured and recorded at any shot produced by the PDS 2000 by the Java Daphne software (Stanghellini and Bortoluzzi 2004) interfaced to a Trimble Acutime and to a Differential Global Positioning System (DGPS). The hull-mounted 16 transducer Benthos Chirp system was used. The data flow and performance were controlled through the Communication’s Technology SWANPRO software. The Subbottom Chirp workstation received positions through a sentence by the PDS 2000; positions were therefore recorded on the XTF trace headers as latitudes and longitudes of the DGPS antenna.

The instrumental offsets (PDS 2000) are presented in the Table 1 (Marsella et al. 2007b).

**Table 1:** **Instrumental offsets on the ship Urania based on the PDS2000 software**

Position	Across	Along	Height
Reference point	0.00	0.00	0.00
DGPS	1.64	14.30	14.18
MBEAM	0.00	14.36	−4.96
MAHRS	0.00	0.00	−3.40
ECHO SOUNDER 33	5.50	−1.85	−3.80
CHIRP	−1.0	11.80	−4.00
A-FRAME	6.5	−6.70	0.0
STERN	0.0	−30.60	0.0
STRING-1	4.00	−60.30	−60.0
STRING-2	−4.00	−60.30	−60.0
GI-GUN ARRAY	0.0	−60.30	−6.0

The GPS antenna, representing the primary positioning system is located on the point DGPS.

### Multibeam bathymetry

The acquisition of Multibeam bathymetric data was carried out through one workstation interfacing the RESON8160 Multibeam system. The adopted Multibeam system was the 50 kHz, 150° aperture RESON 8160 Multibeam, having a range of 5000 m. The sonar head was positioned on the skip’s keel using a V-shaped steel frame. A sound velocity probe at the sonar head was directly interfaced to the processor of the Multibeam system, thus providing the necessary real time data for the beam forming. In addition, two datasets were generated and stored on separate computer for back-up of data on HD and CD/DVD. The PDS2000 software was able to build a 20 m Digital Terrain Model (DTM) during the acquisition of the bathymetry in the survey area. The existing Multibeam dataset will therefore be used for an up-to-date regional bathymetric compilation.

The calibration of the Multibeam data has been carried out through the acquisition of some lines. Heading and pitch values have been easily found, whereas the roll values have been difficult to obtain due to the roughness of the sea bottom morphology.

### CTD casts

**Figure 8:**
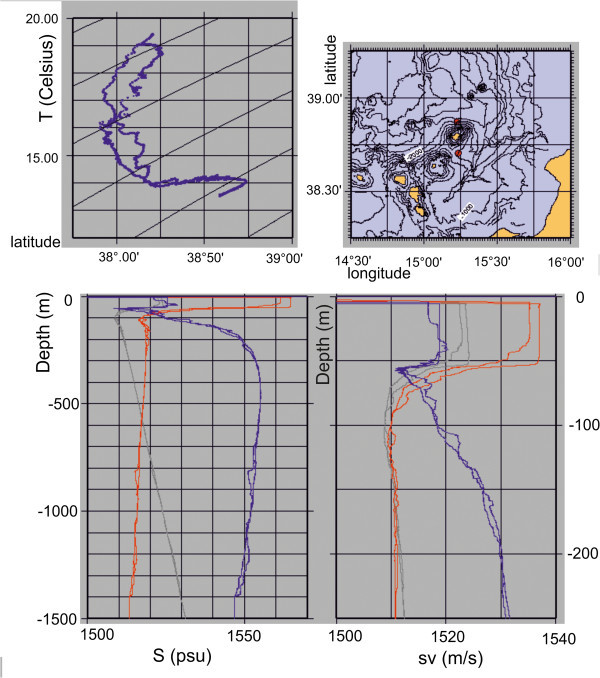
**Data of the CTD cast SBE911 PLUS recorded during the STRO06 oceanographic cruise (modified after Marsella et al.** 2007a **).** The location of the CTD casts acquired in the STRO06 oceanographic cruise is reported in the map on the upper right in the figure. The inset on the lower right of the figure reports the sound velocity diagram (sv, measured in m/s). The inset in the upper left of the figure reports the temperature (T, measured in Celsius). The inset in the lower left of the figure reports the salinity (S, measured in PSU).

The CTD casts were collected in the study area during the acquisition of Multibeam bathymetry. The CTD data were recorded by using a Mod. 911Plus SBE profiling system. The position of the CTD stations is reported in the Table 2. The raw data were recorded and processed through the processing software respectively named SEASAVE and SBEdata. The sound velocity data from the acquired profiles were immediately imported into the PDS2000 software for the Multibeam data corrections. Moreover an example of CTD data collected in this cruise is shown in Figure 8. The location of the CTD stations is reported in the upper right inset of the above mentioned figure (Figure 8).

**Table 2:** **CTD stations positioning in the Multibeam acquisition system**

Station	Data time UTC	Longitude	Latitude
01	2006-11-28 22.25:42	15:13.42	38:52.25
02	2006-12-02 22:38:01	15:14.00	38:42.21

### Subbottom chirp

A largely spaced grid of Subbottom Chirp profiles has been recorded. It has allowed the delimitation of the main units cropping out at the sea bottom, the recognition of the morpho-structures and the calibration of the multibeam geologic interpretation. The seismic grid, radial with respect to the shoreline has been recorded on the continental slope. The location of the seismic profiles has been reported in a sketch navigation map (Figure 1). The map has been constructed using a geographic information system (GIS) importing the files related to the navigation lines recorded onboard by the PDS2000 navigation program. The data processing has been realized using the SEISPRHO software, developed for the elaboration of seismic profiles (Gasperini and Stanghellini 2009). This program processes files recorded in a SEGY format and produces, as a final result, seismic sections as bitmap images. A time variant gain (TVG) has been applied improving the quality of the seismic signal and the visualization of the scattered volcanic sequences.

### Geologic interpretation particularly referred to volcanic geomorphology

The first phase of data processing and interpretation consisted of the cartographic restitution of the Multibeam data as bathymetric maps with contour isobaths and shaded relief maps for the geological interpretation of the main morpho-structural lineaments. The geologic interpretation was carried out based on the recognition of the main morpho-structures cropping out at the sea bottom. The interpretation of high resolution seismic profiles allowed us to reconstruct the structural and stratigraphic setting of the continental slope successions.

The stratigraphic units belong to the Late Quaternary depositional sequence (Catalano et al. 1996; Fabbri et al. 2002). From the Late Pleistocene to the Holocene the space and time evolution and the lateral and vertical migration of the coastal marine, continental shelf and slope depositional environments have been recognized in this sequence. The stratigraphic succession has recorded the variations of the accommodation space of the Late Quaternary deposits during the last 4^th^ order glacio-eustatic cycle, ranging in age between 128 ky (Tyrrhenian stage) and recent times (isotopic stage 5e; Shackleton and Opdyke 1973; Martinson et al. 1987).

The occurrence of outcrops of volcanic acoustic substratum at the sea bottom has not permitted a classical stratigraphic approach applied taking into account the stratigraphic relationships between the acoustic basement and the filling units. The systems tracts of the Late Quaternary depositional sequence, being limited by time-transgressive surfaces, may be considered equivalent to the units bounded by unconformities (Unconformity-Bounded Stratigraphic Units; UBSU; Chang 1975; Nummendal and Swift 1987; Galloway 1989; Sacchi et al. 1999).

The techniques and methods of modern volcanic geomorphology have been adopted and taken into account in the geologic interpretation of the data collected in the Stromboli volcanic island (Grosse et al. 2012; Lahitte et al. 2012; Platz et al. 2012; Procter et al. 2012; Rodriguez-Gonzalez et al. 2012; Thouret 1999; Thouret and Nemeth 2012; Torrecillas et al. 2012).

The significance of volcanic geomorphology has been improved through the quantitative classification of volcanic landforms, which blends morphometry and studies based on ground observations, remote sensing data and laboratory experiments, as well as the diversified use of airborne images and digital data acquired through radar and satellites and combined with the Digital Elevation Models data (Thouret 1999). The monitoring of morphological changes in volcanic areas provides a fundamental contribution to the comprehension of the dynamics of the volcanic systems both during the eruptions and in post-eruptive stages. The rates of geomorphological processes acting at all scales on volcanoes has to be measured to improve the theoretical aspects of volcanic geomorphology (Thouret 1999). The production and comparison of Digital Elevation Models is necessary to document and quantitatively describe the morphological evolution induced by volcanic constructional and destructive processes, such as the emplacement of lava flows, calderic collapses and gravitational instabilities. In particular, for submarine areas, the acoustic techniques as the Multibeam bathymetric surveys allows to investigate the sea floor with an increasing detail and full coverage.

Large scale instability processes, erosional landforms and debris avalanches present a particular relevance in the case study of the Stromboli island. As a consequence of rapid construction, many volcanoes are liable to massive flank or slope failures resulting from structural instability. The slope failures have produced mobile debris avalanches, travelling on long distances beyond the flank of volcanoes at high velocities. A description of geological processes involving the flank failures and the related deposits has been provided in detail by several authors (Voight et al. 1983; Siebert et al. 1987; Crandell 1988; Glicken 1991; Moore et al. 1994).

Massive landslides create specific morphology and deposits, such as horseshoe-shaped re-entraints of the volcanic edifice, such in the case of Ischia island (Chiocci et al. 1998; Aiello et al. 2009, 2012) and high steep escarpments having an amphitheater shape. The debris avalanches typically form a hummocky terrain with water filled depressions and steep flow margins and thick hummocky deposits with block and matrix facies, consisting of unsorted angular-to-subangular debris. A relationship exists between the distance run-out travelled by an avalanche and the failure volume.

As an analytic technique, the volcanic geomorphology can identify the sedimentary facies associations and the facies models for dynamic volcano-sedimentary systems and establish the criteria for recognizing volcaniclastic deposits in old volcanic successions, inferring the role of climatic and tectonic effects on transport and deposition and analyzing the characteristics of sediment gravity flows to determine relevant parameters for modeling their behavior (Thouret 1999).

## Results

### Morpho-bathymetry and geology

The materials erupted by the Stromboli volcano, which shows a volcanic activity constant during geological time, slide on the surrounding slopes and deposit on the submarine flanks of the edifice, which are disrupted by submarine instabilities (gravitative mass fluxes, debris flows, debris avalanches, slides, rock falls, slumpings and erosion along channels). The flanks of the Stromboli island are characterized by the occurrence of lineaments of gravity instability, frequent on the flanks of the volcanic islands (Chiocci et al. 1998). Proceeding eastwards a chute of anastomized detritus occurs, generated by channellised fluxes of detritus organized in transversal bottom lineaments. To the north of the island, channellised fluxes act on a flat sea bottom, characterized by a low reflectivity; to the west it occurs the submarine continuation of the “Sciara del Fuoco”, representing a main lava flow always active, which can be followed for several kilometers before that it laterally joins the Stromboli canyon.

The Stromboli canyon represents one of the most important canyons of the Tyrrhenian sea and links the shoreline of the Sicily with the Tyrrhenian bathyal plain surrounding the Aeolian islands. The canyon receives channellised fluxes on its right side (among them the Gioia canyon) and mass fluxes on its left side (also on the flanks of the Stromboli volcanic island), where its thalweg touches the Aeolian volcanic archipelago.

**Figure 9:**
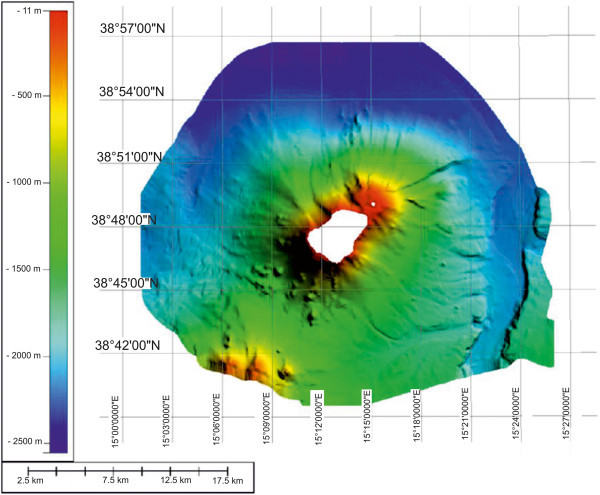
**Shaded relief image map of Stromboli island.** The overall extension of the survey is of about 910 km^2^ in a bathymetric range from 11 m to 2555 m. The Multibeam data give new interesting evidences to understand the morphological, volcanological and structural setting of the Stromboli island, particularly referring to gravity instability processes (lateral collapses on the flanks of the volcanoes).

**Figure 10:**
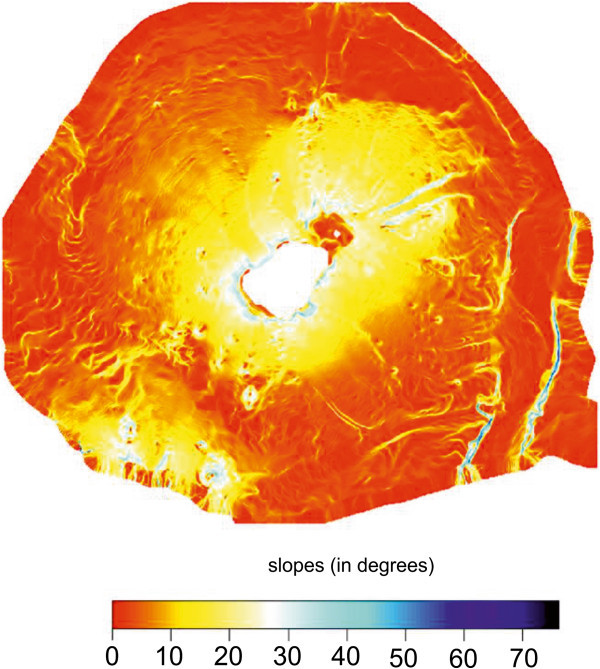
**Slope map of the submerged sector of the Stromboli volcanic edifice.** Some main morpho-depositional volcanic domains have been identified and related to slopes (see the text for further discussion).

A shaded relief image map of the Stromboli island has been constructed and interpreted (Figure 9). The overall extension of the bathymetric survey is of about 910 km^2^ in a bathymetric range from 11 m to 2555 m. The Multibeam data give new interesting evidence to understand the morphological, volcanological and structural setting of the Stromboli island, particularly referring to gravity instability processes, focusing on lateral collapses on the flanks of the volcanoes.The geological interpretation of the Digital Terrain Model of the Stromboli island shows several main morphological lineaments (Figure 9). On the right of the island it is worth noting the occurrence of the Stromboli canyon, fed by a lateral tributary channel, draining the volcaniclastic input coming from the submerged volcanic edifice of the Panarea island. Two main morpho-structural lineaments have been identified, one having a N-S trending and another having NE-SW trending. The submerged flanks of the volcanic edifice are dissected by morpho-structural lineaments controlled by the activity of submarine instability processes along steep slopes. On the upper right side in the Multibeam map it is possible to see the Strombolicchio plateau, located at water depths ranging between 50 m and 150 m isobaths. On the upper left side in the Multibeam map it is worth note the occurrence of the end of the Angitola canyon, draining the volcaniclastic input coming from the continental slope off Calabria.A slope map of the Stromboli volcanic edifice offshore has been constructed based on Multibeam bathymetric data (Figure 10). The slopes of the upper submerged part of the stratovolcano are minor than 10°. A steep break in slope signs the passage to the lower part of the volcanic edifice, showing slopes ranging between 10° and 25°. The last slope gradient occurs in correspondence to the passage from the basal part of the edifice, characterized by slopes ranging between 0° and 5°. Note that the margin of the submerged “sciaras” (lava flows), of the more pronounced channels and of the Stromboli canyon show slopes in the order of 35°-40°.

**Figure 11:**
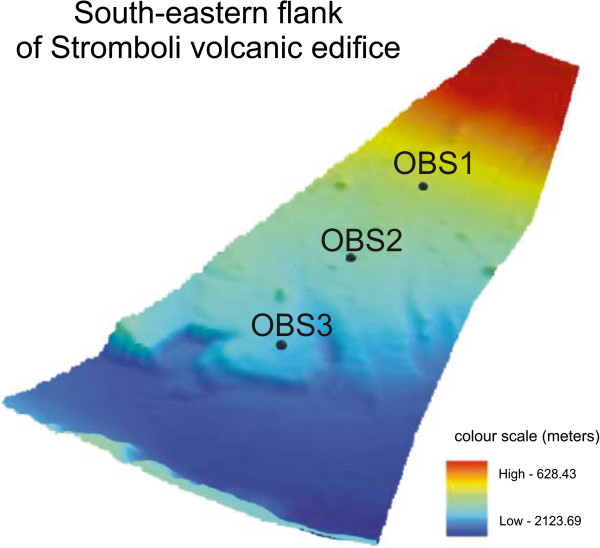
**Detailed shaded relief map of the south-eastern flank of the submerged part of the Stromboli volcano constructed based on Multibeam bathymetry.**

**Figure 12:**
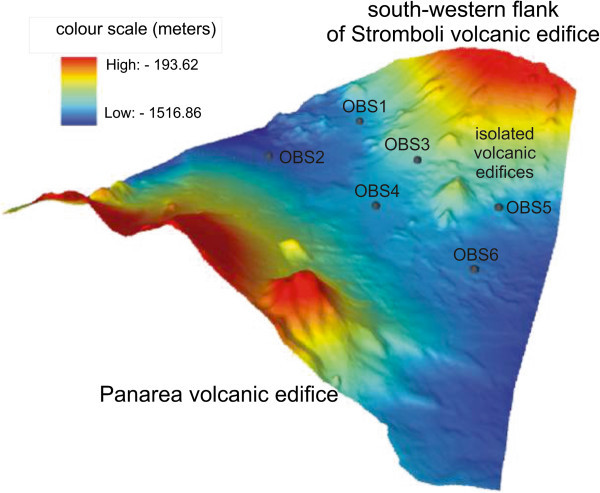
**Detailed shaded relief map of the south-western flank of the submerged part of the Stromboli volcano constructed based on Multibeam bathymetry.**

**Figure 13:**
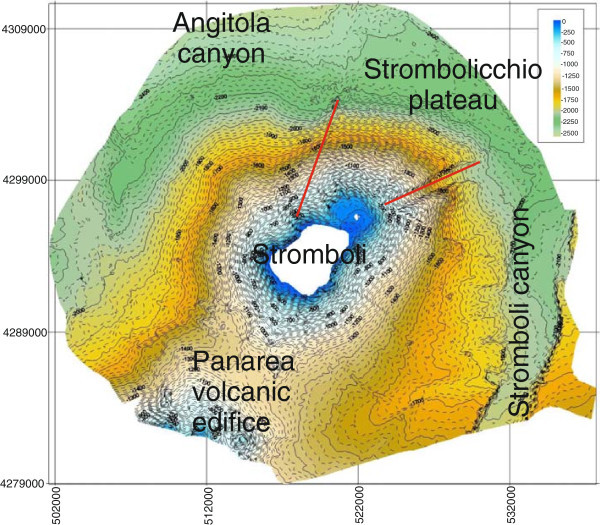
**Shaded relief and contour map of the isobaths of the Stromboli submerged volcanic edifice.**

**Figure 14:**
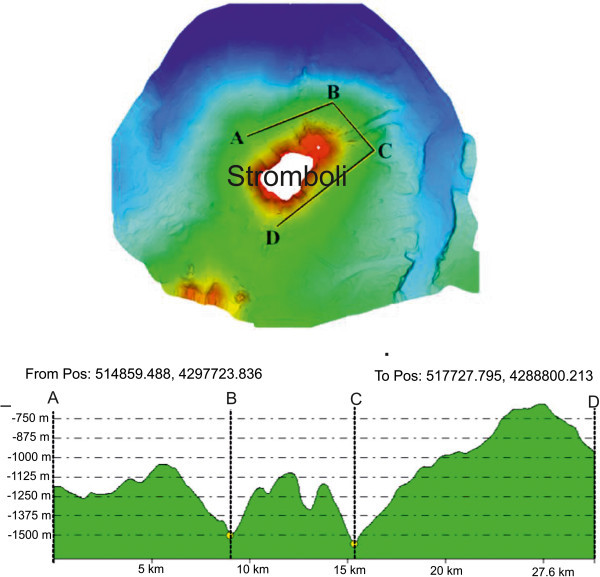
**Bathymetric profile ABCD in the Stromboli offshore and relative location map.** The profile runs in the upper part of the submerged volcanic edifice.

**Figure 15:**
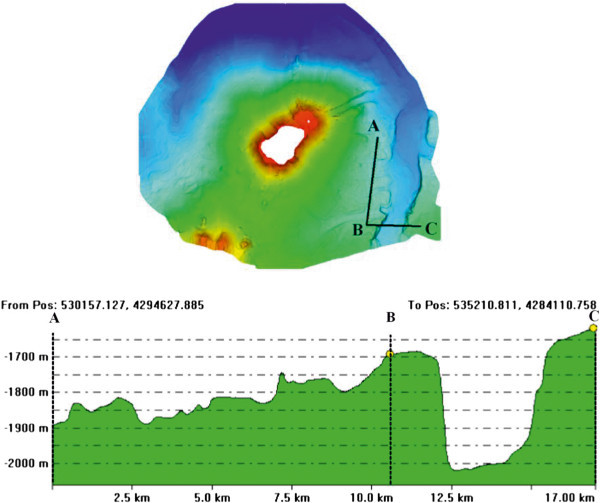
**Bathymetric profile ABC in the Stromboli offshore and relative location map.** The profile runs along the south-eastern flank of the Stromboli volcanic edifice.

Detailed shaded relief maps of the south-eastern and south-western flanks of the Stromboli volcano have been constructed (Figures 11 and 12). The circles indicate the location of the three OBS stations used for a location of a refraction seismic survey carried out during the cruise STRO-06 in a preliminary project phase. It is worth noting the break in slope which signs the passage from the base of the submerged volcanic edifice of the Stromboli island and the bathyal plain, located at around – 2100 m of water depths, at the passage with the volcanic edifice of the Panarea island. In the lower part of the figure a detailed shaded relief map of the south-western flank of the island is reported. The circles indicate the location of six OBS stations positioned before the execution of the wide angle refraction seismics (Marsella et al. 2007a; 2007b). Parasitic vents related to the main volcanic edifice have also to be noted.A shaded relief and contour map of the isobaths of the submerged volcanic edifice has also been constructed (Figure 13). On the right in the map of Figure 13 the Stromboli canyon has been identified, draining the volcaniclastic supply coming from the submerged volcanic edifice of the Panarea island. Two main morpho-structural lineaments, respectively N-S and NE-SW trending have been recognized. The submerged flanks of the volcanic edifice are involved by morpho-structural lineaments controlled by gravitational instabilities along steep slopes (Figure 13). In the upper right of the map the Strombolicchio plateau has been identified at water depths ranging between 50 and 150 m. In the upper left of the figure the lower part of the Angitola canyon has been identified, draining from the volcaniclastic input coming from the Calabria region (Figure 13). The northern flank of the Panarea volcanic edifice has been identified in the lower part of the Figure 13.The constructed bathymetric profile ABCD (Figure 14) runs parallel to the break in slope joining the steep upper part of the submerged volcanic edifice with its lower part, having less steep slopes. The AB segment crosses the submarine prosecution of the “Sciara del Fuoco” lava flow and shows hints of an intense channelization, while the BC segment is comprised between two minima of water depths in the bathyal plain, crossing a deep channel located in the north-eastern flank of the volcanic edifice, having a structural control (Figure 14). The CD segment crosses the south-eastern flank of the submerged volcanic edifice, characterized by submarine gravity instabilities (Figure 14).The constructed bathymetric profile ABC runs along the south-eastern flank of the Stromboli volcanic edifice and crosses the Stromboli canyon (Figure 15). The AB segment shows an overall decrease of water depth proceeding from north to south and hints of several channels with several order of dimensions (Figure 15). The BC segment shows a section of the Stromboli canyon, having a flat thalweg and asymmetrical levees (Figure 15).

### Interpretation of Chirp seismic data

**Figure 16:**
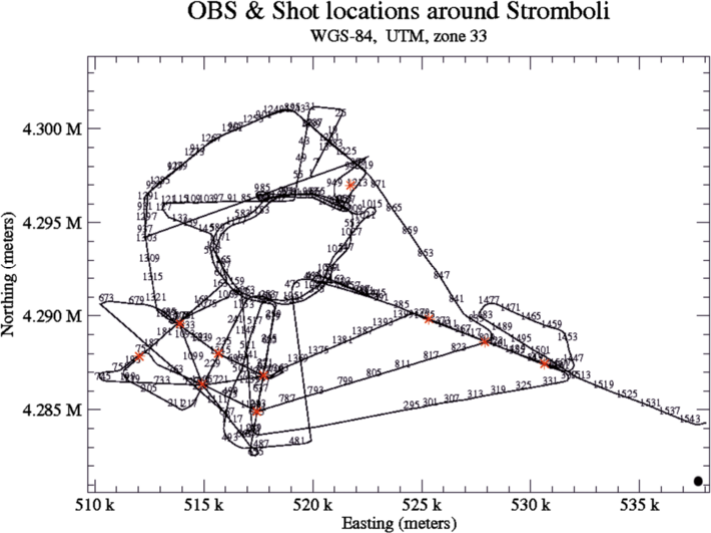
**Sketch map showing the Ocean Bottom Seismometers (OBS) and shot locations around the Stromboli island.**

A sketch map showing the OBS and shot locations around Stromboli has been constructed for a better visualization of the shots of the seismic data collected offshore the island (Figure 16). Some seismic profiles have been processed and interpreted referring to the above mentioned location map. Seismic processing consisted of a ri-lecture of seismic data using the SEISPRHO (software) allowing the generation of bitmap files starting from SGY files of seismic lines (Gasperini and Stanghellini 2009). The processed and interpreted seismic lines are listed in the Table 3.

**Table 3:** **Sketch table reporting the line names, the shot numbers and the seismic source of the collected data**

Line name	Shot numbers	Seismic source
Chirp Stromboli canyon P	0-3118 shots	Subbottom Chirp
Chirp_Stromboli_canyoo	0-6219 shots	Subbottom Chirp
Chirp_Stromboli1_est	0-360 shots	Subbottom Chirp
Chirp_Stromboli1_esu	0-6219 shots	Subbottom Chirp
Chirp_Stromboli1_esv	0-6219 shots	Subbottom Chirp
Chirp_Stromboli1_esw	0-550 shots	Subbottom Chirp
Chirp_Stromboli1_esx	0-962 shots	Subbottom Chirp
Chirp_Stromboli1_esy	0-947 shots	Subbottom Chirp
Chirp_Stromboli_canyon	0-6129 shots	Subbottom Chirp
Chirp_Stromboli_canyos	0-2213 shots	Subbottom Chirp

**Figure 17:**
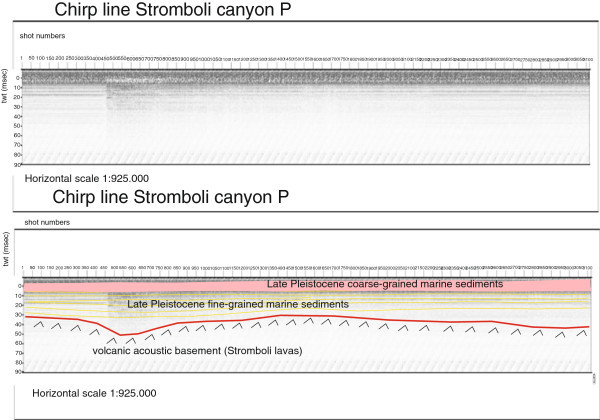
**Chirp line Stromboli canyon P and corresponding geologic interpretation.**

**Figure 18:**
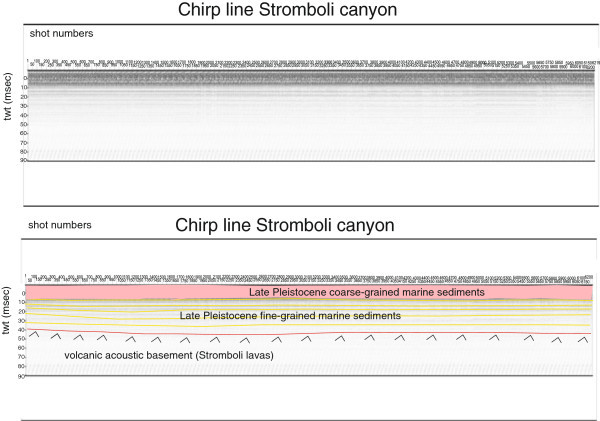
**Chirp line Stromboli canyon and corresponding geologic interpretation.**

**Figure 19:**
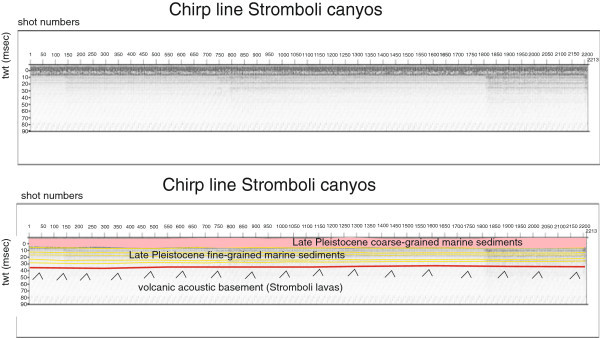
**Chirp line Stromboli canyos and corresponding geologic interpretation.**

**Figure 20:**
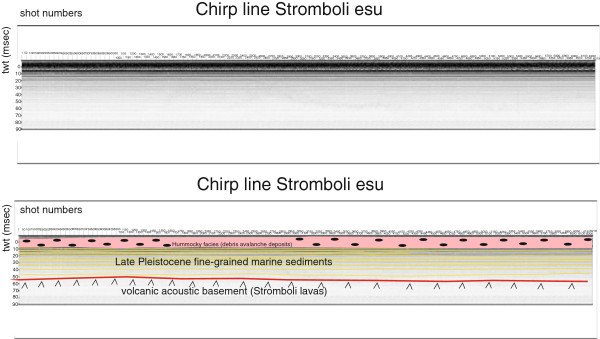
**Chirp line Stromboli esu and corresponding geologic interpretation.**

**Figure 21:**
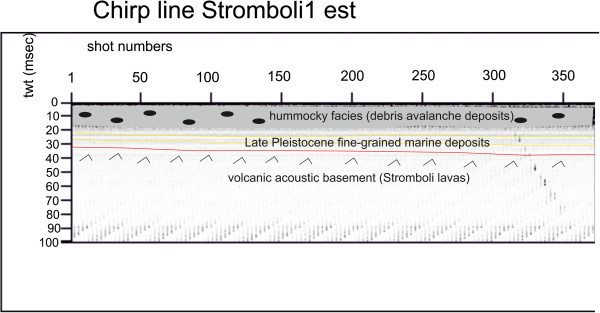
**Chirp line Stromboli 1 est and corresponding geologic interpretation.**

**Figure 22:**
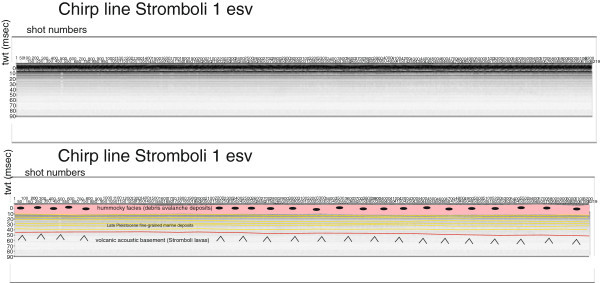
**Chirp line Stromboli 1 esv and corresponding geologic interpretation.**

**Figure 23:**
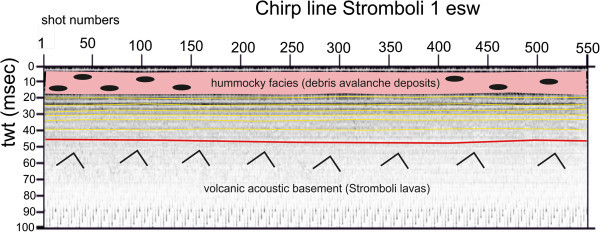
**Chirp line Stromboli 1 esw and corresponding geologic interpretation.**

**Figure 24:**
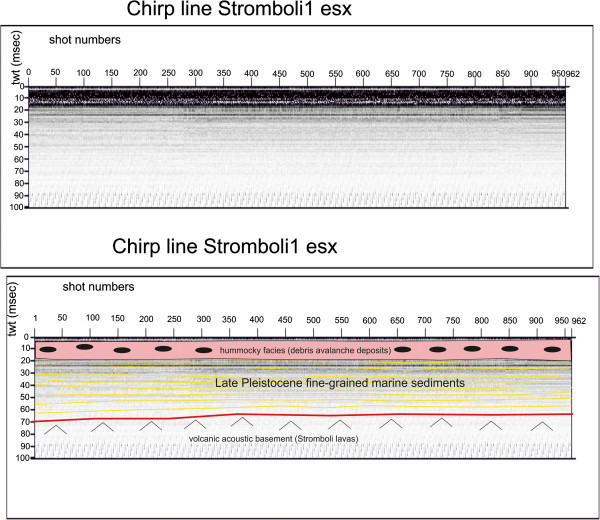
**Chirp line Stromboli 1 esx and corresponding geologic interpretation.**

Several seismic units have been identified based on the geologic interpretation of Subbottom Chirp profiles recorded around the volcanic edifices and interpreted as volcanic acoustic basement pertaining to the volcano and overlying slide chaotic bodies emplaced during its complex volcano-tectonic evolution. They are related to the eruptive activity of Stromboli, mainly poliphasic and to regional geological processes involving the geology of the Aeolian Arc.The interpretation of the Chirp line Stromboli canyon P (Figure 17) has suggested the occurrence of a volcanic acoustic basement, genetically related to the Stromboli lavas, located at depths among 30 and 60 msec. This basement is overlain by a relatively thick sequence, characterized by parallel seismic reflectors and interpreted as Late Pleistocene fine-grained sediments (Figure 17). This sequence is overlain by another sedimentary sequence, recognized up to the sea bottom and characterized by a scattered seismic signal of high amplitude. It has been interpreted as Late Pleistocene coarse-grained marine sediments.The interpretation of the Chirp line Stromboli canyon (Figure 18) has confirmed the stratigraphic setting seen in the above mentioned profile. A volcanic acoustic basement has been identified at an average depth of 40 msec. This basement is covered by a seismic sequence, characterized by parallel reflectors and interpreted as Late Pleistocene fine-grained sediments (Figure 18). This sequence is overlain by another seismic sequence, occurring up to the sea bottom and characterized by a scattered seismic signal having a high amplitude. The sequence has been already interpreted as Late Pleistocene coarse-grained marine sediments. Similar results have been obtained through the interpretation of the Chirp line Stromboli canyos (Figure 19).The interpretation of the Chirp line Stromboli esu has evidenced the occurrence of a volcanic acoustic basement overlain by a seismic sequence interpreted as Late Pleistocene marine coarse-grained sediments (Figure 20). In the first subbottom a thick seismic sequence characterized by hummocky facies has been interpreted as debris avalanche deposits genetically related to the Stromboli volcano (Figure 20). Similar results have been obtained through the geologic interpretation of the Chirp line Stromboli 1 est (Figure 21) and of the Chirp line Stromboli 1 esv (Figure 22). The same seismic sequences have been recognized also on the seismic lines Stromboli 1 esw and Stromboli 1 esx (Figures 23 and 24).

## Concluding remarks

Some new insights about the morpho-bathymetry, marine geology and seismic stratigraphy of the Stromboli submarine area have been discussed through Multibeam and seismic data. New bathymetric maps are here presented coupled to seismic interpretation in order to provide new data on the submarine structure of the volcanic edifice of the Stromboli island.

The formation of the Aeolian Arc and consequently of the Stromboli island, is genetically related to the effect of the passive roll-back of the subduction plan of the Ionian crust under the Calabria region (Maliverno and Ryan 1986; Kastens et al. 1988) and to geodynamic processes of partial melting at the scale of the mantle (Marani and Trua 2002; Figure 4), allowing for the eastward migration of regional extension and creation of oceanic crust in the Southern Tyrrhenian bathyal plain (Savelli 1984; Rehault et al. 1987; Sartori et al. 1986, 1987; Panza et al. 2003; Procter and Sheridan 2012).

Along the Tyrrhenian margin of Southern Italy and northern Sicily it has been noted a wide seismicity characterized by shallow hypocenters, while in the Southern Tyrrhenian sea middle and deep earthquakes have been detected. According to the interpretations of some authors the hypocenters define a Benioff zone, suggesting the occurrence of a subduction plan arcuated with the concavity towards the north-west (Gasparini et al. 1982; Chouet, 1985; Chouet et al. 2003). The deep seismicity zone of the southern Tyrrhenian is overlain by the recent volcanic arc of the Aeolian islands (Barberi et al. 1978; Beccaluva et al. 1985), which is part of a more extended system of submerged volcanic seamounts (Enarete, Eolo, Alcione, Lametini, Palinuro). The magmatic alimentation both for the Aeolian volcanic islands and for the Marsili back-arc basin derives from the lateral flux of deep asthenosphere around the Ionian slab and to the consequent fertilization and partial melting of the mantle wedge (Marani and Trua 2002). The common magmatic source both for the arc and the back-arc is supported by the observation that the lavas sampled on the Marsili volcano are comparable, from a compositional point of view with the lavas of insular arcs typical of the Aeolian volcanoes (Beccaluva et al. 1985).

Eight subaerial volcanic edifices are located in correspondence to the Aeolian Arc. While the most part of these volcanoes are supplied by fluid magmas genetically related to the Ionian lithospheric slab, other ones (Vulcano, Lipari and Salina) are aligned along a regional strike-slip fault having a NNW-SSE trending, cutting also the Etna volcano. The submarine volcanic districts of the Tyrrhenian sea have been recently analyzed also as possible geothermal resources, considered the high values of heat flow related to these volcanic structures (Signanini et al. 2006). Recent Multibeam surveys of the Southern Tyrrhenian sea have furnished morphological observations of great detail on the structure of the submerged and emerged volcanic arc and on the Tyrrhenian bathyal plain (Marani and Gamberi 2004a, 2004b).

In the Aeolian Arc the volcanic activity was explicated with four main phases, ranging in age from 1–1.3 My B.P. at the Sisifo seamount and at the Filicudi volcanic island (Beccaluva et al. 1985). From 0.8 My B.P. to recent times shoshonitic and calcalkaline lavas, consisting of basalts, andesitic basalts and rhyolites have been erupted in the volcanic complexes, both subaerial and submarine (Finizola et al. 2003); (Beccaluva et al. 1985). The volcanic edifice of the Stromboli island started to form about 110 ky ago. The volcano shows symmetric flanks and a conical shape and has an average elevation of about 927 m above sea level. It represents the emerged part of an important volcanic edifice, high more than 3000 m. The eruptive activity, typically poliphasic, has controlled a stratigraphic architecture characterized by overlapping of different volcanic products (lavas and pyroclastites). The formation of the volcano started about 200 ky B.P. in the north-eastern sector of the island with the growth of a volcanic edifice now completely eroded, whose central neck is represented by the Strombolicchio inlet. About 100 ky B.P., in correspondence to the present-day volcanic edifice, a new volcano started to grow (Paleostromboli I), reaching a height of 400 m; a great part of this volcano was downthrown after great explosions, leaving at its place a caldera having an elliptical shape. The caldera depression was then infilled by the growth of a new volcano, reaching the height of 700 m (Paleostromboli II). The life of this volcano concluded about 35 ky ago with the downthrowing of a new caldera, having a circular shape. About 34 ky B.P. a new volcano, called Vancori underwent a giant sliding in its upper part and in the western flank. To testify this collapse a large amphitheater remains, which nowadays surrounds the present top of the Stromboli volcano, including the active crater.

The structure of the Stromboli volcanic island has been recently related to that one of the Campania volcanoes: in both ones a well-developed low velocity layer, having a thickness of 10–15 km occurs under a thin lid, overlain by a thin continental crust. The structural difference among the Stromboli volcano and the proximal volcanoes of Volcano and Lipari is confirmed by the different geochemical characters (Finizola et al. 2003; Revil et al. 2004).

The collected geological data well fit with the results of the paper of Kidd et al. (1998) focusing on the marine geological setting of the Aeolian islands and Stromboli canyon. The basin margins are characterized by slump scars, channels and large debrites on the continental slope off Calabria region, not imaged by the data discussed in the present paper. Blocky hummocky avalanche deposits have been recognized on the flanks of the Stromboli volcano (Kidd et al. 1998). This latter evidence is substantially in agreement with seismo-stratigraphic data shown in the Subbottom Chirp lines interpreted in this paper, referring in particular to the Chirp line Stromboli esu (Figure 20), Stromboli 1 est (Figure 21), Stromboli 1 esv (Figure 22), Stromboli 1 esw (Figure 23) and Stromboli 1 esx (Figure 24). In the Stromboli canyon and in minor deep sea channels sediment transport by turbidity currents generates sediment waves. Between the basin margins and the abyssal plain, the outcropping volcanic basement traps part of the sediment coming from the marginal area (Kidd et al. 1998). The volcanic acoustic basement genetically related to the Stromboli lavas has been widely recognized also in the seismic lines shown in this paper, referring in particular to the Chirp lines Stromboli canyon P (Figure 17), Stromboli canyon (Figure 18) and Stromboli canyos (Figure 19). Moreover, the abyssal plain surrounding the volcanic edifice is characterized by low relief lobes and ponded sediments (Kidd et al. 1998). Circular high backscatter patches have been recognized through the interpretation of Sidescan Sonar photomosaics (Kidd et al. 1998), indicating that volcanic blocks have been transported downslope in the Stromboli canyon.

Blocky hummocky facies have also been recognized in the seismic lines interpreted in the present paper. The streaked high backscatter patterns at the scarp base are interpreted as coarse-grained sediments transported downslope along the Stromboli canyon (Kidd et al. 1998). Coarse-grained facies have also been recovered in the seismic lines shown in this paper.
